# Later aorta operation after aortic valve replacement for bicuspid aortic valve

**DOI:** 10.1186/s13019-024-02638-6

**Published:** 2024-03-19

**Authors:** Kohei Hachiro, Noriyuki Takashima, Tomoaki Suzuki

**Affiliations:** Division of Cardiovascular Surgery, Department of Surgery, Shiga University of Medical Science, Setatsukinowa-cho, Otsu, 520-2192 Shiga Japan

**Keywords:** Later aorta operation, Bicuspid aortic valve, Aortic valve replacement

## Abstract

**Background:**

We investigated long-term outcomes, particularly later aorta operations and overall death in patients who underwent aortic valve replacement for bicuspid aortic valve without aortic surgery.

**Methods:**

Between January 2002 and December 2022, 274 patients underwent aortic valve replacement for bicuspid aortic valve at our institution. Of them, 181 patients who did not undergo aortic surgery, in accordance with current guidelines, were analyzed retrospectively.

**Results:**

The median follow-up duration was 6.1 (2.0–10.6) years, and follow-up was completed in 97.8% of pateints. There were 3 patients (1.7%) who underwent later aorta operation during follow-up period. The cumulative later aorta operation rate at 10 years adjusting overall death as competing risk was 16.3%, and the estimated rates of freedom from overall death at 10 years was 83.7%. Fine-Gray competing risk regression model showed that aortic valve stenosis was only the predictor of later aorta operation (hazard ratio 8.477; *p* = 0.012). In multivariable Cox models, predictors of overall death were aortic valve stenosis (hazard ratio: 8.270, 95% confidence interval: 1.082–63.235; *p* = 0.042) and operation time (hazard ratio: 1.011, 95% confidence interval: 1.004–1.017; *p* = 0.002).

**Conclusions:**

Patients with bicuspid aortic valve with ascending aortic diameter less than 45 mm are at low risk of later aorta operation after isolated aortic valve replacement.

## Introduction

Surgical intervention is recommended for ascending aorta diameter (AAD) of 45 mm or more during a concomitant aortic valve replacement (AVR) for bicuspid aortic valve (BAV) [1, 2]. The development of bicuspid aortopathy has been attributed to hemodynamic and genetic factors [3–6]. Magnetic resonance imaging studies have shown that an RL fusion pattern (fusion between right and left coronary cusps) causes a flow jet directed toward the right anterior aortic wall [7, 8]. The increase in regional wall shear stress has been thought to be the basis of the association between the RL fusion pattern and dilation of the aortic root and asymmetric dilation of the ascending aorta. On the other hand, an RN fusion pattern (fusion between right and non-coronary cusps) causes a flow toward the posterior aorta, which increases wall shear stress at the right posterior aspect of the aorta. Additionally, abnormal processing of the extracellular matrix protein fibrillin 1 by vascular smooth-muscle cells causes detachment of vascular smooth-muscle cells from the extracellular matrix, which leads to the release of matrix metalloproteinases together with their tissue inhibitors [3, 4, 6, 9, 10]. The resulting matrix disruption and elastin and lamellar fragmentation result in increased apoptosis of vascular smooth-muscle cells and disruption of the media layer, which adversely affect the structural integrity and flexibility of the aorta.

AVR eliminates those hemodynamic factors, but not any gene-related factors. Therefore, there may be more concern about the risk of long-term aortic problems following bicuspid valve replacement than there would be after replacement of a tricuspid aortic valve. However, the rate of later aorta operation in patients who underwent AVR for BAV without aortic resection remains unclear. In the current study, we investigated long-term outcomes in patients undergoing isolated AVR for BAV in accordance with current guidelines.

## Patients and methods

Informed consent was obtained from all patients to use their medical records for research purposes, and the ethics committee of Shiga University of Medical Science approved this study (Reg. No. R2022-218; approval date: March 24, 2023).

Between January 2002 and December 2022, 274 patients underwent AVR for BAV at our institution. We excluded 83 patient who underwent concomitant aortic surgery and 10 patients who underwent AVR for infectious endocarditis. Finally, 181 patients were included in the study, and we retrospectively investigated perioperative and long-term outcomes.

### Outcome measures and definitions

The primary outcome was later aorta operation and the secondary outcome was overall death. We defined cardiac death as deaths caused by myocardial infarction, heart failure or lethal arrhythmia. Cause of death was collected from witnesses, family members, death certificates, hospital records and autopsy records.

### Surgical treatment

In accord with current guidelines [2, 11], we performed concomitant ascending aorta replacement in each patient whose AAD was 45 mm or more at the time of AVR for BAV. Valves, selected by each surgeon’s preference, were implanted in the supra-annular position or intra-annular position at each surgeon’s preference, too. After completion of AVR, aortomy was sutured using 4 − 0 monofilament continuous suture in 2 layers, or 4 − 0 monofilament horizontal mattress suture and continuous suture. In our cohort, one patient underwent minimally invasive cardiac surgery through a right minithoracotomy and all others underwent a median sternotomy. Myocardial protection was provided using antegrade or retrograde cold blood cardioplegia.

### Follow-up details

The patients underwent annual echocardiographic follow-up at our institution, and computed tomography (CT) examination was performed according to the judgment of the outpatient doctor. In the follow-up period, ascending aorta replacement or total arch replacement was performed if the thoracic aorta diameter reached 55 mm or larger.

### Statistical analysis

Continuous variables with normal distribution are presented as mean ± standard deviation and those with non-normal distribution are presented median and interquartile range. Categorical variables are presented as percentages. We estimated probabilities of survival using the Kaplan–Meier method, in which patients still alive were censored at the date of their last follow-up. The cumulative later aorta operation rate was calculated adjusting overall death as competing risk. We performed univariable and multivariable Cox proportional hazards regression analyses to analyze overall deaths. Fine-Gray competing risk regression model was developed to estimate the risk of later aorta operation adjusting overall death as competing risk. Variables reaching a *P* value of < 0.050 in the univariable analysis, or those which were considered clinically important, were used into the multivariable model. All statistical analyses were two-sided, and results were considered statistically significant in which P was < 0.050. We performed all statistical analyses using SPSS, version 29.0 (IBM Corp., Armonk, NY) and SAS, version 9.4 (SAS Institute, Cary, NC).

## Results

The mean age of our study population was 66.8 ± 11.6 years, and 124 (68.5%) were males (Table 1). There were 146 (80.7%) patients who underwent AVR for aortic valve stenosis (AVS). There were 87 patients whose coronary cusps fusion pattern was RL pattern.

**Table 1:** Preoperative Patient Characteristics

Age (year)	66.8 ± 11.6
Sex (male)	124 (68.5%)
Body mass index (kg/m^2^)	22.5 ± 3.4
Hypertension	92 (67.5%)
Diabetes mellitus	36 (19.9%)
Dyslipidemia	52 (28.7%)
Smoking history	89 (49.2%)
Previous CVD	8 (4.4%)
Previous PCI	4 (2.2%)
Creatinine (mg/dL)	0.84 (0.70–1.03)
Aortic valve lesion	
Stenosis	146 (80.7%)
Regurgitation	35 (19.3%)
RL fusion pattern	87 (48.1%)
LV ejection fraction (%)	57.1 ± 12.6
LV end-diastolic diameter (mm)	53.5 ± 9.3
LV end-systolic diameter (mm)	37.3 ± 9.6
Ascending aorta diameter (mm)	39.1 ± 3.9

CVD: cerebrovascular disease; LV: left ventricular; PCI: percutaneous coronary intervention

### Early outcomes

Table 2 shows operative and postoperative outcomes. The mean operation time was 214 ± 56 min. There were 137 patients who underwent AVR using bioprosthetic valve, and the mean valve size was 23.7 ± 2.2 mm. There were 24 patients who underwent coronary artery bypass grafting, 13 underwent mitral valve repair, 5 underwent mitral valve replacement, and 9 underwent tricuspid valve repair. One patient suffered a postoperative stroke, and one underwent reoperation for bleeding. Hospital mortality and 30-day mortality were zero.

**Table 2:** Operative and Postoperative Data

Operative data	
Operation time (min)	214 ± 56
Cardiopulmonary bypass time (min)	105 ± 28
Aortic clamp time (min)	72 ± 15
Bioprosthetic valve	137 (75.7%)
Valve size (mm)	23.7 ± 2.2
Cannulation	
Ascending	169 (93.4%)
Femoral	9 (5.0%)
Axillary	3 (1.7%)
Concomitant procedures	
Coronary artery bypass grafting	24 (13.3%)
Mitral valve repair	13 (7.2%)
Mitral valve replacement	5 (2.8%)
Tricuspid valve repair	9 (5.0%)
Postoperative data	
Stroke	1 (0.6%)
Deep sternal wound infection	0 (0%)
Reoperation for bleeding	1 (0.6%)
ICU stay > 48 h	5 (2.8%)
Ventilation > 48 h	3 (1.7%)
30-day mortality	0 (0%)
Hospital mortality	0 (0%)

ICU: intensive care unit

### Long-term outcomes

**Fig. 1:**
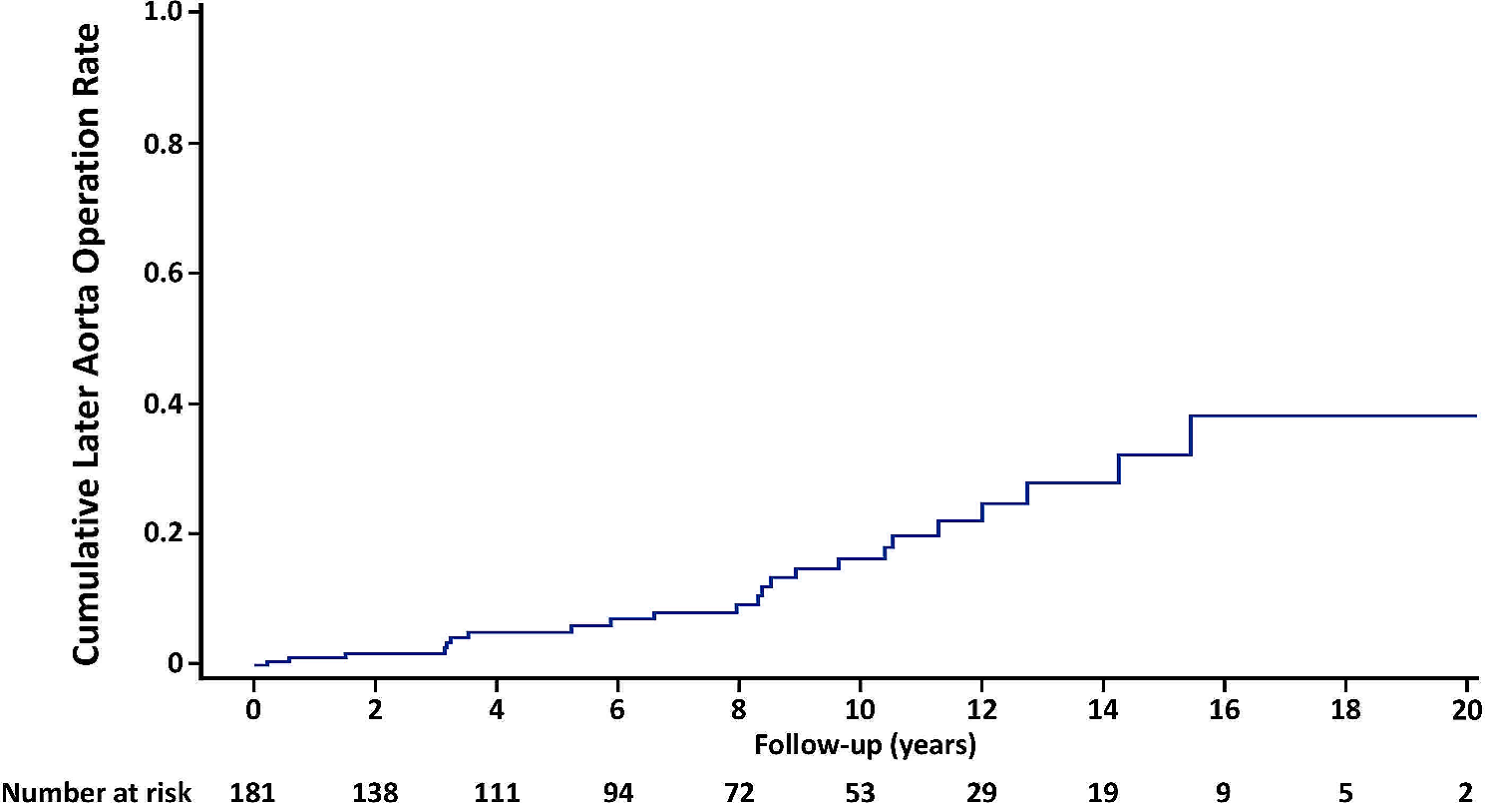
Cumulative Later Aorta Operation Rate

Follow-up was completed in 97.8% of patients (177/181), and the median follow-up duration was 6.1 (2.0–10.6) years (maximum: 20.2 years). There were 54 (29.8%) patients who underwent CT as outpatients every year. In our cohort, 3 patients (1.7%) underwent aortic reoperation in the follow-up period. One female patient underwent AVR for AVS when she was 72 years old as first surgery, and preoperative CT showed her AAD was 44.6 mm. She underwent total arch replacement 7.1 years later because her AAD progressively dilated to more than 50 mm. Another female patient underwent AVR for AVS at age 68 years, and then ascending aorta replacement 10.2 years later because her AAD dilated to 53 mm. Her preoperative CT at first surgery showed an AAD of 44.5 mm. One male patient underwent AVR for AVS at age 68 years, when preoperative CT showed an AAD of 44.8 mm. He underwent emergency ascending aorta replacement for acute type A aortic dissection 14.5 years later. In that case, primary entry was in the middle of the ascending aorta; he had been seen annually as an outpatient, and the most recent CT had shown an AAD of 52.0 mm. The cumulative later aorta operation rate at 10 years was 16.3% (Fig. 1). All of them did not have family history of aortopathy. No patient underwent endovascular aortic repair during follow-up period. Fine-Gray competing risk regression model showed that AVS was only the predictor of later aorta operation (hazard ratio [HR] 8.477; *p* = 0.012).

**Fig. 2:**
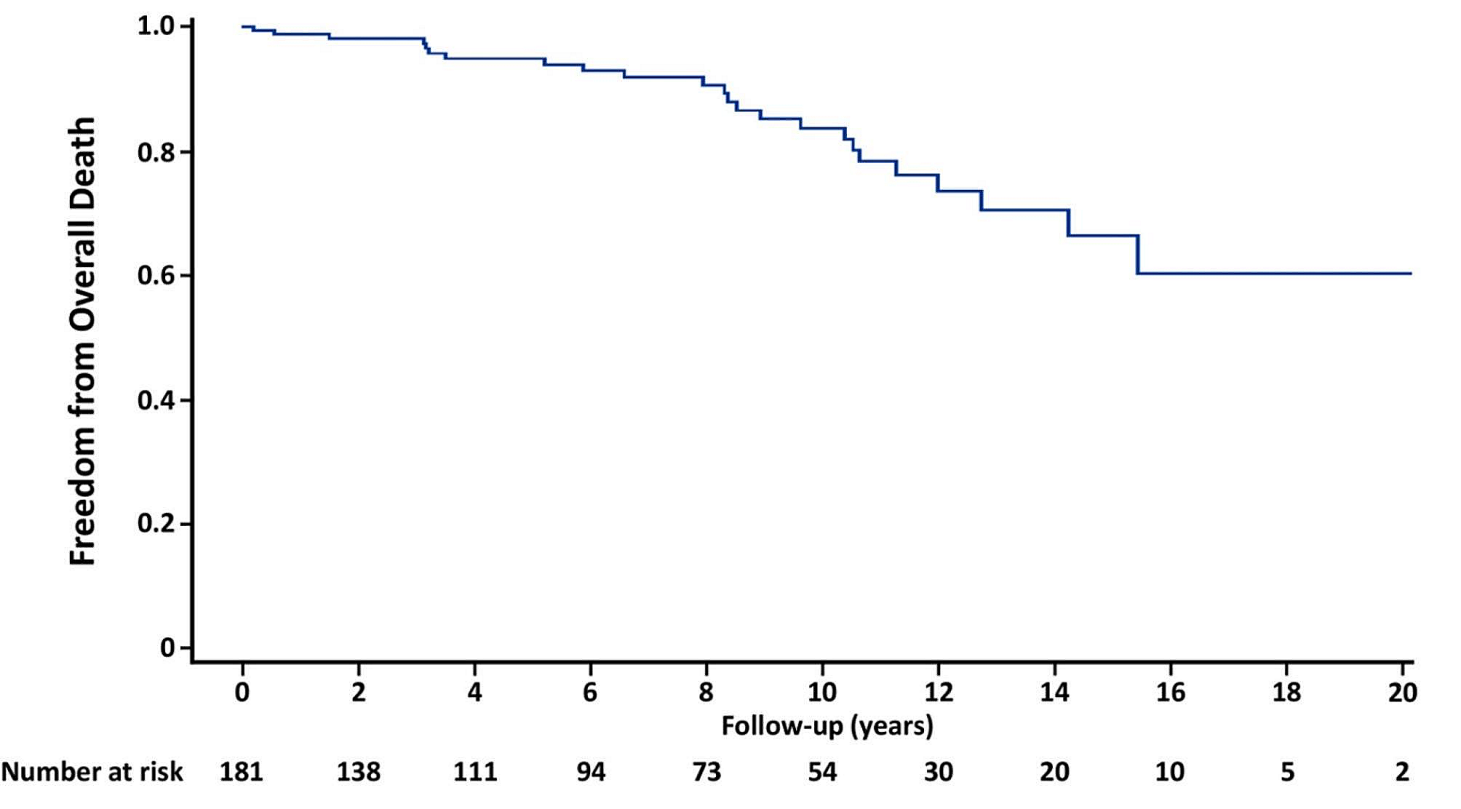
Freedom from Overall Death

There were 24 patients who died during the follow-up period. Table 3 shows all causes of death. 2 patients died of cardiac events: one died 10.6 years after AVR and the other died 11.3 years after AVR; both died of heart failure. No patient died because of acute aortic disease such as acute aortic dissection and aortic aneurysm rupture. The adjusted 10-year estimated rate of freedom from all-cause death was 83.7% (Fig. 2). Multivariable analysis for overall death showed that the independent predictors were AVS (HR 8.270; 95% confidence interval [CI] 1.082–63.235; *p* = 0.042) and operation time (HR 1.011; 95% CI 1.004–1.017; *p* = 0.002) (Table 4).

**Table 3:** Causes of Overall Death

Cardiac death	2 (1.1%)
Myocardial infarction	0 (0%)
Heart failure	2 (1.1%)
Lethal arrhythmia	0 (0%)
Noncardiac death	22 (12.2%)
Acute aortic disease	0 (0%)
Pneumonia	1 (0.6%)
Stroke	1 (0.6%)
Sepsis	1 (0.6%)
Cancer	7 (3.9%)
Unknown	12 (6.6%)

**Table 4:** Multivariable Cox Proportional Hazards Model for the Predictors of Overall Death

Predictor	HR	95% CI	*P* value
Sex (male)	3.812	0.812–17.888	0.090
Diabetes mellitus	1.372	0.553–3.404	0.496
Smoking history	1.737	0.634–4.761	0.283
Creatinine (mg/dL)	1.077	0.888–1.307	0.451
Aortic stenosis	8.270	1.082–63.235	0.042
Operation time	1.011	1.004–1.017	0.002

CI: confidence interval; HR: hazard ratio

In our cohort, two patients underwent repeat aortic valve surgery. They both underwent trans-catheter valve implantation for structural valve deterioration, one 11.3 years later, and one 18.4 years later.

## Discussion

Previously, we reported the risk factors for dilation of the aorta over time after AVR for BAV which focused on the possible impact of valve fusion pattern [12]. In that study, the presence of aortic regurgitation and AAD > 40.0 mm at time of surgery were shown as significant predictors of dilation of the aorta after AVR, but valve fusion pattern did not. In this study, we investigated the actual long-term results, including the rate of reoperation related to the aorta.

The rate of later aorta operation after AVR for BAV remains unclear. Our major finding of the current study was that the cumulative rate of later aorta operation rate at 10 years was 16.3% (Fig. 1). This result does not indicate a need to perform concomitant ascending aorta replacement at the time of AVR for BAV if AAD is below 45 mm, as recommended in the current guidelines [2, 11]. Girdauskas and associates compared the risk of late aortic events after isolated AVR surgery for bicuspid versus tricuspid aortic valve stenosis with concomitant ascending aortic dilatation of 40 to 50 mm [13]. In 153 patients diagnosed with bicuspid aortic valve stenosis, 5 patients (3.3%) required later aorta operation and aortic dissection did not occur in the BAV group within follow-up period (mean 11.5 years) in their study, which was comparable to our results. However, in the present study, all the three patients (1.7%) who underwent reoperation during the follow-up period had preoperative AAD more than 44.0 mm, so patients with larger AAD at the time of AVR for BAV seems to be needed careful follow-up monitoring of aorta diameter.

Existing American College of Cardiology/American Heart Association and European Society of Cardiology guidelines do not specify a treatment strategy for the thoracic aorta in patients who have undergone AVR for BAV previously [2, 11]. AVR for BAV can eliminate hemodynamic factors but not genetic factors. Aortic problems may therefor occur sooner in patients who underwent AVR for BAV than in patients who underwent AVR of a tricuspid aortic valve. In fact, in the present study, one patient underwent emergency surgery for acute type A aortic dissection, who had had a CT follow-up as an outpatient every year. His most recent AAD before developing acute type A aortic dissection was 52.0 mm. The fact that the entry was located in the middle of the ascending aorta, not at the site where arterial line was inserted, also suggests that the aorta was fragile. In the ACC/AHA guidelines, surgery is indicated in patients with aneurysm of the aortic root or ascending aorta who have a maximum diameter of 55.0 mm or more (Class 1), and in patients with aneurysm of the aortic root or ascending aorta who have a maximum diameter of 50.0 mm or more when performed by experienced surgeons in a Multidisciplinary Aortic Team (Class 2a) [11]. In the ESC guidelines, surgery is indicated in patients with no elastopathy with ascending aortic aneurysm of 55.0 mm or more (Class 2a) and in patients with a bicuspid valve with risk factors with ascending aortic aneurysm of 50.0 mm or more (Class 2a) [2]. In a patient who has undergone AVR for BAV thoracic aorta surgery may be necessary before dilation to 55 mm, for example, at 50 mm.

In our institution, AAD in echocardiography results means the diameter of the ascending aorta in the long-axis view of the left ventricle. However, the maximum diameter of ascending aorta is often located in a more distal portion than that seen on the long-axis view. In our cohort, the mean preoperative AAD in echocardiography results was 32.6 ± 4.6 mm. On the other hand, the mean preoperative AAD in CT was 39.1 ± 3.9 mm. Therefore, it is problematic to measure the maximum diameter of ascending aorta with echocardiography alone. Considering the possible genetic factors, we may need to follow up with not only echocardiography but also CT every year in all patients who underwent AVR for BAV.

### Study limitations

This study had several limitations. First, the sample size of the present study was small. There were only 3 patients who underwent aortic reoperation during the follow-up period, which may be associated with less statistical power. Second, all our cohorts included Japanese patients only, that may limit generalizability. Third, we could not know the cause of death in 12 patients. Of them, some patients may have died because of valve or aorta related event. Finally, only 29.8% in our cohort underwent CT as outpatients every year. Therefore, we could not completely follow aortic diameter after AVR for BAV.

## Conclusions

Patients with BAV with AAD less than 45 mm are at low risk of later aorta operation after isolated AVR, which suggest that there is no need to lower the criterion for aorta replacement at the time of AVR below the current standard of 45 mm.

## Data Availability

The data that support the findings of this study are available from the corresponding author, [K. Hachiro], upon reasonable request.

## Abbreviations

AAD: ascending aorta diameter
AVR: aortic valve replacement
AVS: aortic valve stenosis
BAV: bicuspid aortic valve
CI: confidence interval
CT: computed tomography
HR: hazard ratio
